# Low Concentrations of Metformin Selectively Inhibit CD133^+^ Cell Proliferation in Pancreatic Cancer and Have Anticancer Action

**DOI:** 10.1371/journal.pone.0063969

**Published:** 2013-05-08

**Authors:** Shanmiao Gou, Pengfei Cui, Xiangsheng Li, Pengfei Shi, Tao Liu, Chunyou Wang

**Affiliations:** Pancreatic Disease Institute, Department of General Surgery, Union Hospital, HUST, Wuhan, P. R. China; University of Pécs Medical School, Hungary

## Abstract

Pancreatic cancer is the fourth leading cause of cancer related deaths in the United States. The prognosis remains dismal with little advance in treatment. Metformin is a drug widely used for the treatment of type II diabetes. Recent epidemiologic data revealed that oral administration of metformin is associated with a reduced risk of pancreatic cancer, suggesting its potential as a novel drug for this disease. Many studies have demonstrated the *in vitro* anticancer action of metformin, but the typically used concentrations were much higher than the *in vivo* plasma and tissue concentrations achieved with recommended therapeutic doses of metformin, and low concentrations of metformin had little effect on the proliferation of pancreatic cancer cells. We examined the effect of low concentrations of metformin on different subpopulations of pancreatic cancer cells and found that these selectively inhibited the proliferation of CD133^+^ but not CD24^+^CD44^+^ESA^+^ cells. We also examined the effect of low concentrations of metformin on cell invasion and *in vivo* tumor formation, demonstrating *in vitro* and *in vivo* anticancer action. Metformin was associated with a reduction of phospho-Erk and phospho-mTOR independent of Akt and AMPK phosphorylation. CD133^+^ pancreatic cancer cells are considered to be cancer stem cells that contribute to recurrence, metastasis and resistance to adjuvant therapies in pancreatic cancer. Our results provide a basis for combination of metformin with current therapies to improve the prognosis of this disease.

## Introduction

Pancreatic cancer is among the most aggressive of solid malignancies. Each year, 43,920 patients are newly diagnosed with the disease, resulting in 37,390 deaths per annum in the United States and making pancreatic cancer the fourth leading cause of cancer related death in both males and females [1]. There has been little advance in treatment and the prognosis remains dismal [2], [3], [4], [5], with a 5 year survival rate of only about 3% and a median survival of less than 6 months. Among patients who undergo potentially curative resection, 5 year survival is less than 24% because of local recurrence and metastasis [1], [6], [7]. Novel therapeutic strategies are therefore urgently needed for this highly malignant disease.

Metformin is a drug widely used for the treatment of type II diabetes. Recently, epidemiologic data revealed that metformin, but not other antidiabetic drugs, decreases the incidence of pancreatic cancer in patients with diabetes mellitus [8], [9]. Interestingly, there was no correlation between the protective effect and patients’ blood sugar levels [9]. A protective effect was also observed in a fat hamster tumorigenesis model of pancreatic cancer using N-nitrosobis-(2-oxopropyl) amine [10]. Several *in vitro* studies have established a direct action of metformin on many types of cancer cells, including those of pancreatic cancer [11], [12]. Metformin may therefore be a potential therapeutic agent in the treatment of pancreatic cancer, though its mechanism of anticancer action is ambiguous. *In vitro* experiments have revealed a dose dependent effect of metformin on cancer cell proliferation. The typically used concentrations in such studies are 5–30 mM, which are much higher than the plasma and tissue concentrations measured in individuals who have received recommended therapeutic doses, and less than 1 mM of metformin has little effect on cancer cell proliferation [13], [14].

Here, we show that low concentrations of metformin have effects on different subpopulations of pancreatic cancer cells according to their differential expression of surface markers. CD133^+^ and CD24^+^CD44^+^ESA^+^ cells are considered pancreatic cancer stem cells, and the proliferation of CD133^+^ but not CD24^+^CD44^+^ESA^+^ cells was selectively inhibited by low concentrations of metformin. Metformin was associated with reductions of phospho-Erk and phospho-mTOR independent of Akt and AMPK phosphorylation. Although low concentration metformin had no effect on the proliferative capacity of pancreatic cancer cells in general, their *in vitro* invasive capacities and *in vivo* pancreatic cancer xenograft growth were significantly inhibited.

## Materials and Methods

### Cell culture

We obtained AsPC-1 and SW1990 cells from the American Type Culture Collection. AsPC-1 pancreatic adenocarcinoma cells were derived from the ascites of a 62-year-old Caucasian female patient with pancreatic adenocarcinoma; SW1990 pancreatic adenocarcinoma cells were derived from metastasis in the spleen of a 56-year-old Caucasian male patient with pancreatic adenocarcinoma. Both cell types were grown in Dulbecco’s modified Eagle medium (DMEM) (Invitrogen, Carlsbad, CA) supplemented 10% fetal bovine serum (FBS) (Gibco, Billings, MT) and penicillin/streptomycin (Invitrogen) at 37°C with 5% CO_2_.

### Flow cytometry

For surface marker detection, cells were resuspended in 100 µL Hank’s balanced salt solution with 1% FBS (Gibco). For isolation of CD133^+^ cells for western blot analysis, cells were resuspended in 100 µL Hank’s balanced salt solution with 1% FBS. Fc Receptor Binding Inhibitor (eBioscience, Inc., San Diego, CA) was added and the sample was incubated for 5 min at 4°C. After two washes, Anti-CD133 fluorescein isothiocyanate (FITC) (Biorbyt, Cambridge, UK), Anti-CD24 FITC (eBioscience), Anti-CD44 PE-Cy5 (eBioscience) or Anti-ESA PE (eBioscience) was added and the sample was incubated for 30 min at 4°C. After two washes, the proportions of subpopulation cells that expressed the different surface markers were determined using a FACSCalibur system (BD Biosciences, San Jose, CA) and cell sorting of CD133^+^ cells was done using a FACSAria system (BD Biosciences). Side scatter and forward scatter profiles were used to eliminate cell doublets.

For apoptosis analysis, cells were treated for 48 h with metformin (0.2 mM for AsPC-1, 0.1 mM for SW1990) or without metformin. First, samples were incubated with Fc receptor binding inhibitor for 5 min at 4°C, then Anti-CD133 FITC was added and the sample was incubated for 30 min at 4°C. After two washes, Annexin V APC and propidium iodide labeling was performed for flow cytometry, which was conducted using an Annexin V assay kit according to the manufacturer’s instructions.

For cell cycle analysis, cells were treated for 48 h with metformin (0.2 mM for AsPC-1, 0.1 mM for SW1990) or without metformin. After fixing the cells in 70% methanol, Fc receptor binding inhibitor was added and the sample was incubated for 5 min at 4°C. Anti-CD133 FITC was then added and the sample was incubated for 30 min at 4°C. After two washes, the sample was treated with RNase and exposed to propidium iodide for flow cytometry.

### Cell proliferation assay

Cell proliferation assays were conducted using CCK-8 according to the manufacturer’s instructions. Cells were seeded into a 96-well plate and cultured in 100 µL of DMEM supplemented with 10% FBS. After 24 h, the seeded cells were treated with 0.02, 0.05, 0.10, 0.20 or 0.50 mM metformin added to the culture medium, or did not receive metformin. At the indicated time points, the medium was exchanged for 110 µL DMEM with CCK-8 reagent and the cells were incubated for 2 h. Absorbance was measured for each well at a wavelength of 450 nm using an auto-microplate reader.

### Growth curve

First, cells were cultured in serum-free DMEM for 12 h. The cells were then detached by trypsinization and plated into six-well plates in DMEM supplemented with 10% FBS with metformin (0.2 mM for AsPC-1, 0.1 mM for SW1990) or without metformin at a concentration of 1×10^3^ cells/well. Cell number was assessed following trypsinization; cell samples were counted on a hemocytometer at 24 h intervals. The results were plotted as a growth curve.

### Cell invasion assay

A cell invasion assay was performed in a 24-well Transwell chamber (Corning, Inc., Corning, NY). First, the 8 µm pore polycarbonate membrane insert was coated with 100 µL of Matrigel (BD Biosciences). Before the assay was performed, cells were treated with metformin (0.2 mM for AsPC-1, 0.1 mM for SW1990) for 48 h or not given metformin. The chambers were then placed in 24-well plates; 1×10^4^ cells in DMEM supplemented with 0.2% FBS were plated into each upper chamber, and DMEM supplemented with 10% FBS with metformin (0.2 mM for AsPC-1, 0.1 mM for SW1990) or without metformin was added to the lower chambers. After incubation at 37°C for 48 h, cells that had invaded to the opposite side of the membrane surface were stained with crystal violet.

### Xenograft experiment

Female *nu*/*nu* mice were obtained from the Experimental Animal Center of Union Hospital, Wuhan, China. For each experiment, mice were randomly distributed into equal groups (four mice per group) that were untreated or treated with metformin. For treated groups, 800 mg/L of metformin was diluted in their drinking water each day for the duration of the experiment; 72 h later, the whole population of pancreatic cancer cells were injected into the right flank of each mouse. The tumors were measured every 3 days after the initial injection and tumor volume (V) was calculated according to V  =  (length × width^2^)/2. The protocol was approved by the Committee on the Ethics of Animal Experiments of the Union Hospital, Huazhong University of Science and Technology (Permit Number: 2009-096).

### Western blotting

Flow cytometry sorted cells were washed in PBS and resuspended in RIPA buffer, 1 mM PMSF, 1 mM Na_3_VO_4_, and 1× protease inhibitor cocktail for 3 min on ice. The lysate was centrifuged at 14,000 × g for 15 min at 4°C and the supernatant was used for western blotting. Protein lysates were boiled in loading buffer (Beyotime, Jiangsu, China), resolved by electrophoresis on 8% SDS-polyacrylamide gels, and transferred to PVDF membranes (Amersham Pharmacia Biotech, Amersham, UK). Membranes were probed overnight at 4°C with AMPKα, phospho-AMPKα (Thr172), Akt, phospho-Akt (Thr308), Erk1/2, phospho-Erk (Thr202/Tyr204), mTOR, and phospho-mTOR (Ser2448) primary antibody (Cell Signaling, Danvers, MA), with β-actin (Cell Signaling) as the control. Horseradish peroxidase-conjugated IgG (Beyotime) was used to detect specific proteins. Finally, immunodetection was conducted using chemiluminescent substrates (Amersham Pharmacia Biotech).

### Statistical analysis

For flow cytometry and cell invasion assays, experiments were performed in triplicate. The xenograft experiment was performed in quadruplicate. For cell proliferation assays and growth curves, experiments were performed in sixes. Data were presented as the mean ± standard deviation, analyzed by one-way analysis of variance and then compared among groups using unpaired Student’s *t*-test. A significance threshold of *P*<0.05 was used. Data were analyzed using SPSS v.11 statistical software (SPSS, Inc.).

## Results

### Low concentrations of metformin did not inhibit proliferation of pancreatic cancer cells

**Figure 1:**
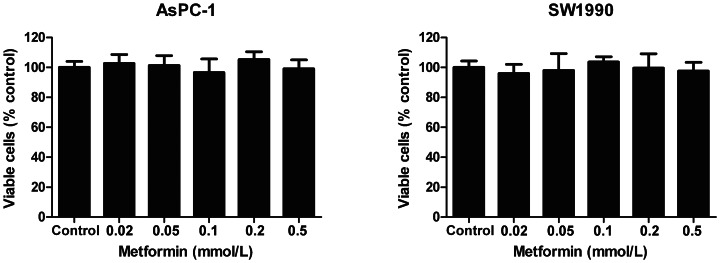
Low concentrations of metformin did not inhibit the proliferation of pancreatic cancer cells. AsPC-1 and SW1990 cells were incubated with different concentrations of metformin for 72 h and numbers of viable cells were determined by CCK-8 assay. The results are presented as the proportion of viable cells relative to the control. No difference in viable cells was observed between cells treated with low concentrations of metformin (≤0.5 mM) and controls. Error bars represent the standard deviation.

To investigate the effect of low concentrations of metformin on the proliferation of pancreatic cancer cells, we conducted a CCK-8 assay using AsPC-1 and SW1990 cells. Metformin has been shown to have little effect on the proliferation of pancreatic cancer cells at low concentrations. As shown in Fig. 1, in the present study cells were treated with 0.01–0.2 mM metformin for 72 h, but their survival was not inhibited.

### Low concentrations of metformin decreased proportion of CD133^+^ cells

**Figure 2:**
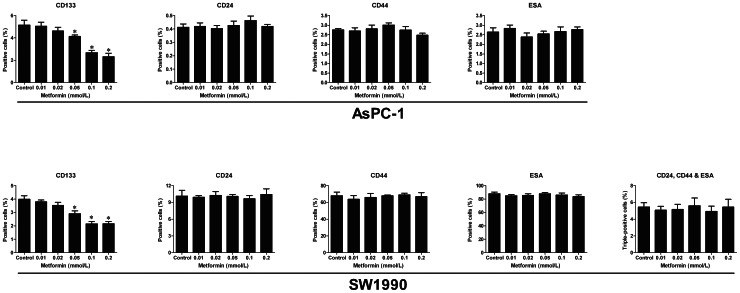
Low concentrations of metformin selectively decreased the proportion of CD133^+^ cells among pancreatic cancer cells. AsPC-1 and SW1990 cells were incubated with different concentrations of metformin for 72 h and the proportions of cells expressing different surface markers were determined by flow cytometry. The results are presented as the proportions of the different subpopulations of cells. The proportions of CD24^+^, CD44^+^, ESA^+^ and CD24^+^CD44^+^ESA^+^ cells (detectable only in SW1990 cells) were not altered by treatment with low concentrations of metformin (≤0.2 mM). The proportion of CD133^+^ cells was reduced in a dose-dependent manner; 0.2 mM metformin for AsPC-1 cells and 0.1 mM metformin for SW1990 cells decreased the proportion of CD133^+^ cells by half. Error bars represent the standard deviation. ^*^ *P*<0.05 (Compared with control)

To investigate the effect of low concentrations of metformin on the proliferation of different subpopulations of pancreatic cancer cells, we conducted a flow cytometry assay using AsPC-1 and SW1990 cells. The cells were treated with 0.01–0.2 mM metformin for 72 h and their expression of surface markers analyzed. As shown in Fig. 2, low concentrations of metformin decreased CD133^+^ cells in a dose dependent manner, but did not affect CD24^+^, CD44^+^, ESA^+^ or CD24^+^CD44^+^ESA^+^ cells (CD24^+^CD44^+^ESA^+^ cells are not detectable among AsPC-1 cells).

### Low concentrations of metformin inhibited proliferation of CD133^+^ cells by G1/S arrest

**Figure 3:**
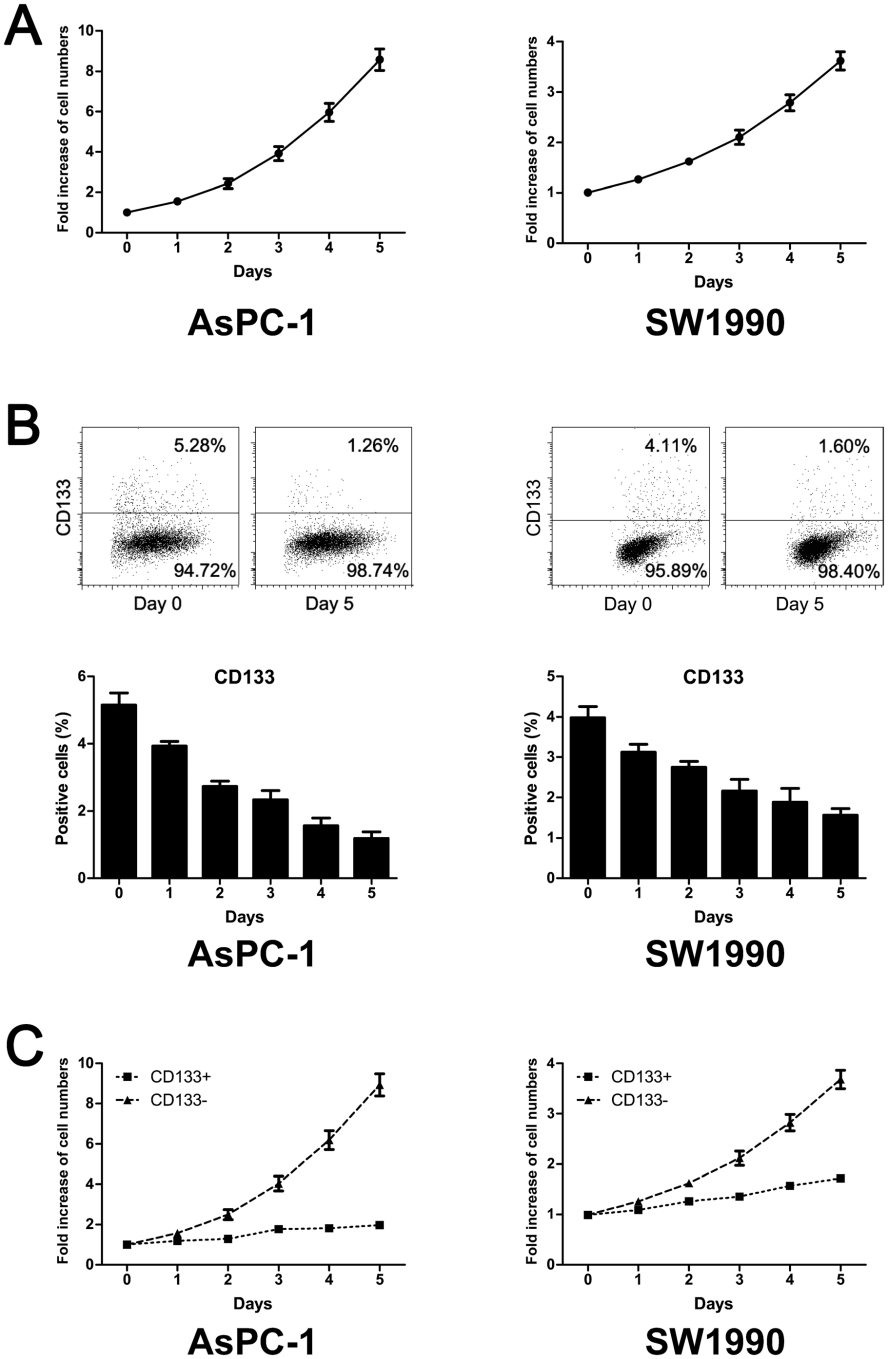
Low concentrations of metformin selectively inhibited the proliferation of CD133^+^ pancreatic cancer cells. A, Growth curves for pancreatic cancer cells treated with low concentrations of metformin. AsPC-1 and SW1990 cells were incubated with 0.2 mM or 0.1 mM metformin, respectively, and their numbers counted at each time point. The results are presented as the fold increase relative to 0 h. B, Effect of low concentrations of metformin on the proportion of CD133^+^ cells. AsPC-1 and SW1990 cells were incubated with 0.2 mM or 0.1 mM metformin, respectively, and the proportion of CD133^+^ cells was determined at each time point. The proportion of CD133^+^ cells decreased gradually. C, Growth curves for CD133^+^ and CD133^–^ pancreatic cancer cells treated with low concentrations of metformin. The total number of pancreatic cancer cells and the proportions of CD133^+^ and CD133^–^ cells were determined at each time point. The results are presented as the fold increase relative to 0 h. The proliferation of CD133^+^ cells was selectively inhibited. Error bars represent the standard deviation.

**Figure 4:**
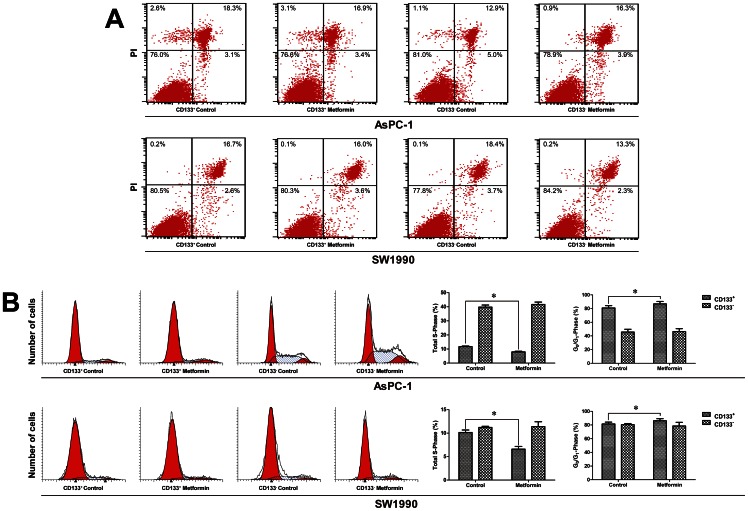
Low concentrations of metformin induced cell cycle arrest but not apoptosis in CD133^+^ pancreatic cancer cells. A, Effect of low concentrations of metformin on apoptosis in pancreatic cancer. AsPC-1 and SW1990 cells were incubated with 0.2 mM or 0.1 mM metformin, respectively, for 72 h and the proportions of apoptotic cells were determined by flow cytometry. No significant differences were observed between the treated cells and controls. B, Effect of low concentrations of metformin on the cell cycle in pancreatic cancer. AsPC-1 and SW1990 cells were incubated with 0.2 mM or 0.1 mM metformin, respectively, for 72 h and the proportions of cells in each stage of the cell cycle were determined by flow cytometry. A decrease of total S phase cells and an increase of G0/G1 phase suggest G1/S arrest in CD133^+^ cells. Error bars represent the standard deviation. ^*^ *P*<0.05.

To investigate the effect of low concentrations of metformin on the proliferation of CD133^+^ pancreatic cancer cells, AsPC-1 and SW1990 cells were treated with 0.2 mM or 0.1 mM metformin, respectively. Cell numbers were counted every 24 h and flow cytometry was conducted to determine the numbers and proportions of CD133^+^ and CD133^−^ cells at the indicated time points. As shown in Fig. 3, the proliferation of CD133^+^ cells was selectively inhibited. Apoptosis and cell cycle analysis were performed 48 h after cells were treated with metformin (0.2 mM for AsPC-1, 0.1 mM for SW1990) or without metformin; these low concentrations induced apoptosis in neither CD133^+^ nor CD133^+^ cells, but the cell cycle of the CD133^+^ cells was altered by the treatment. Low concentration metformin increased the proportion of CD133^+^ cells in G0/G1 phase and decreased that of cells in S phase significantly. The CD133^–^ cell cycle was not affected (Fig. 4).

### Low concentrations of metformin inhibited invasion of pancreatic cancer cells

**Figure 5:**
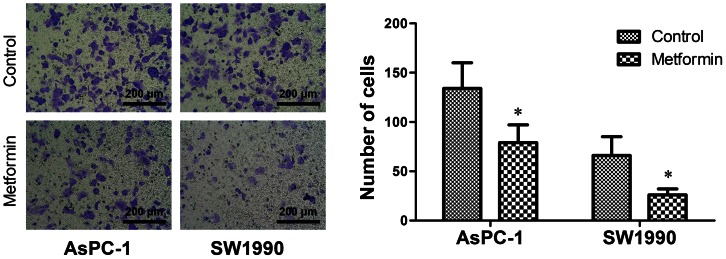
Low concentrations of metformin inhibited the invasion of pancreatic cancer cells. Effect of low concentrations of metformin on invasion in pancreatic cancer. AsPC-1 and SW1990 cells were incubated with 0.2 mM or 0.1 mM metformin, respectively, for 72 h and cell invasion was determined by Transwell assay. Low concentrations of metformin reduced the invasion of pancreatic cancer cells. Error bars represent the standard deviation. ^*^ *P*<0.05.

A Transwell assay was conducted to examine the effect of low concentrations of metformin on pancreatic cancer cell invasion. Treatment with metformin (0.2 mM for AsPC-1, 0.1 mM for SW1990) decreased the number of cells that invaded to the opposite side of the membrane of the Transwell chamber compared with cells that did not receive metformin (Fig. 5B), which suggests that low concentration metformin inhibits the invasive capacity of pancreatic cancer cells.

### Oral administration of metformin inhibited pancreatic cancer xenograft growth in vivo

**Figure 6:**
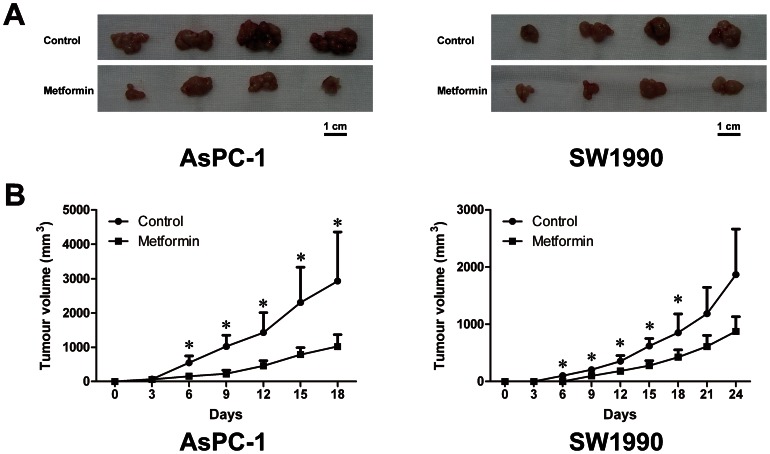
Oral administration of metformin inhibited pancreatic cancer xenograft growth. A, Xenograft at sacrifice. Eight hundred milligrams per liter of metformin was diluted in the drinking water of *nu*/*nu* mice 72 h before injection of pancreatic cancer cells. Mice were sacrificed 18 or 24 days after the implantation. Xenografts from mice treated with oral metformin were much smaller than those from untreated mice. B, Effect of oral administration of metformin on growth of xenografts. Eight hundred milligrams per liter of metformin was diluted in the drinking water of *nu*/*nu* mice 72 h before injection of pancreatic cancer cells. Tumors were measured every 3 days after the injection and tumor volume (V) was calculated according to V =  (length × width^2^)/2. Xenograft growth was significantly inhibited by oral administration of metformin. Error bars represent the standard deviation. ^*^ *P*<0.05.

To investigate the effect of low dose metformin on pancreatic cancer *in vivo*, xenograft experiments using *nu*/*nu* mice were conducted. For mice treated with metformin, the amount of drug diluted in their drinking water was equivalent to a human dose of 20 mg/kg by normalization to surface area; the plasma concentration of metformin in the mice was about 0.02 mM. Mice were sacrificed 18 (AsPC-1 cells) or 24 (SW1990 cells) days after they were injected with pancreatic cancer cells (5×10^6^ for AsPC-1 cells, 1×10^7^ for SW1990 cells). The growth of pancreatic cancer xenografts was significantly inhibited by metformin treatment (Fig. 6).

### Low concentrations of metformin inhibited phosphorylation of mTOR independent of Akt and AMPK phosphorylation in CD133^+^ cells

**Figure 7:**
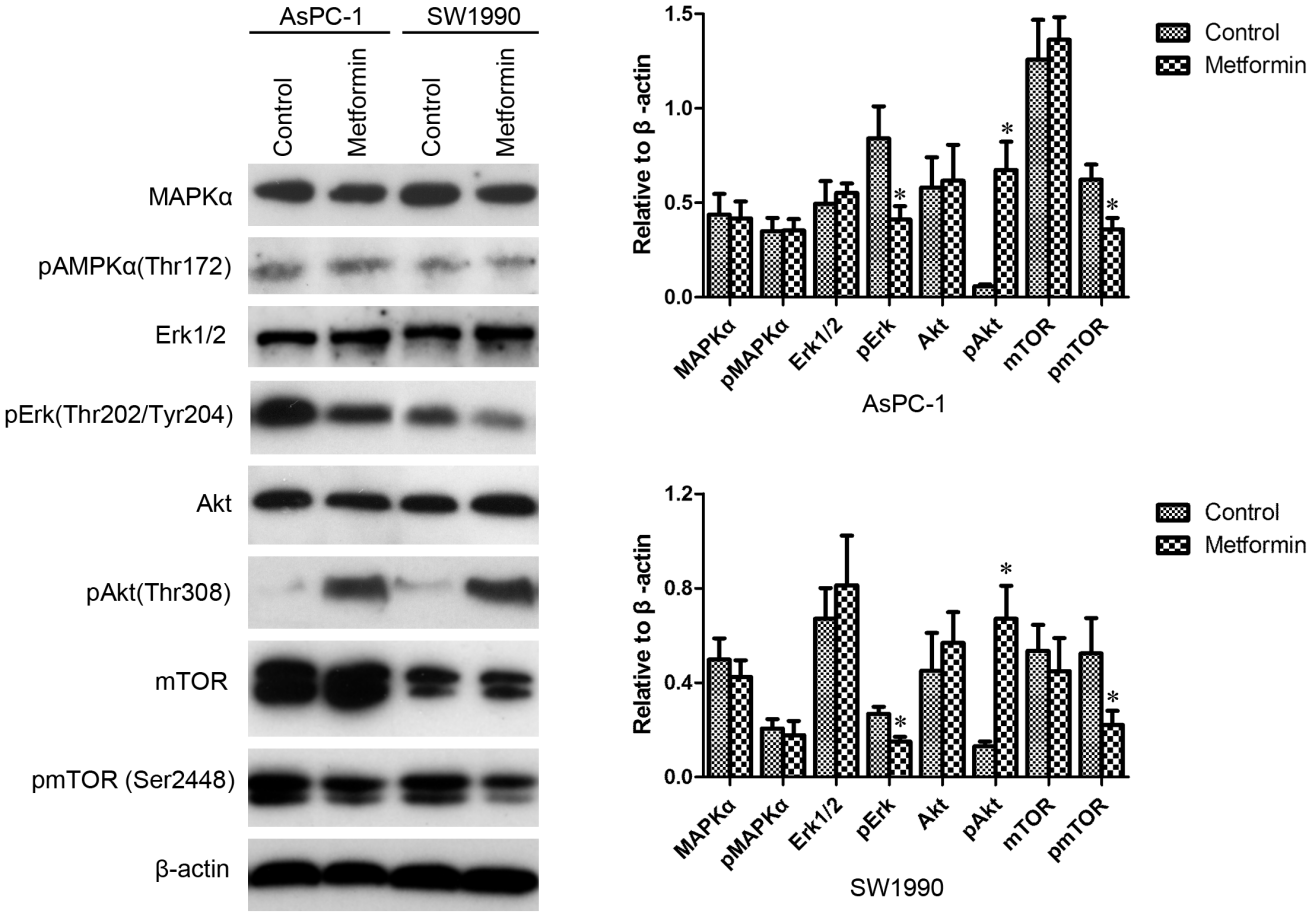
Low concentrations of metformin inhibited Erk and mTOR phosphorylation and increased Akt phosphorylation of CD133^+^ pancreatic cancer cells. Pancreatic cancer cells were treated with metformin for 4 h (0.2 mM for AsPC-1, 0.1 mM for SW1990) and CD133^+^ cells were sorted by flow cytometry. Expression of AMPKα, Akt, Erk1/2, and mTOR and phosphorylation of CD133^+^ cells were evaluated by western blotting and the results were quantified using ImageJ V.1.46r (National Institutes of Health). Significant decreases of phospho-ERK1/2 and phospho-mTOR expression and a significant increase of phospho-Akt expression were observed in the metformin treated cells. Error bars represent the standard deviation. ^*^ *P*<0.05.

To identify possible molecular determinants of the effects of metformin on CD133^+^ cells, we evaluated the activation of AMPK, Erk, and Akt – three kinases that are possibly involved in these effects. mTOR, which is phosphorylated by AMPK, Erk, and Akt, was also evaluated. After treatment with metformin for 4 h (0.2 mM for AsPC-1, 0.1 mM for SW1990), reductions of phospho-Erk and phospho-mTOR were observed in CD133^+^ cells, suggesting a requirement for inhibition of Erk and mTOR by the antiproliferation action of metformin. No significant change of phospho-AMPKα was observed, whereas phospho-Akt increased after treatment with metformin, which suggests that the inhibitory effect of metformin on mTOR was independent of AMPK and Akt phosphorylation (Fig. 7).

## Discussion

Metformin reduces hepatic glucose production and increases insulin sensitivity and glucose utilization by muscles and adipocytes, resulting in decreased insulinemia and amelioration of insulin sensitivity in diabetic patients. It is the most frequently prescribed antidiabetic drug for type II diabetes [15] and is also used for other diseases that feature insulin resistance, including polycystic ovary syndrome [16], non-alcoholic fatty liver disease [17] and premature puberty [18]. Recently, it has gained attention for its potential efficacy as an anticancer drug. In pioneering work, Evans et al. first demonstrated in 2005 that taking metformin may be associated with a reduced risk of cancer in patients with type II diabetes [19]. In 2009, a case control study focusing on the effect of antidiabetic therapies on the risk of pancreatic cancer was published by Li et al. and demonstrated that metformin significantly decreased the risk of pancreatic cancer, with an odds ratio of 0.38 [9]. The efficiency of metformin in decreasing the incidence of pancreatic cancer has been supported by other epidemiologic and animal studies [8], [10].

Although metformin’s antidiabetic mechanism of action remains uncertain, activation of AMP-activated protein kinase (AMPK) has been widely accepted as a possible mechanism. AMPK is an important enzyme involved in insulin signaling, the metabolism of glucose and fats, and glucose production by liver cells. Many cellular studies have focused on AMPK and related molecules and have revealed an anticancer action of metformin *in vitro* [12], [20], [21]. A recent study also demonstrated the antitumor action of metformin on pancreatic cancer stem cells [13]. Of note, most of these experiments used concentrations of metformin (typically 5–30 mM) that are much higher than the recommended therapeutic doses for clinical use. When concentrations were decreased to the same order as that found in the plasma and tissues of individuals receiving therapeutic doses, inhibition of cell proliferation was not observed. Our data show that proliferation of pancreatic cancer cells is not inhibited by less than 0.5 mM metformin.

Most tumors, including pancreatic cancer, are heterogeneous; that is, they comprise cells of varying phenotypic and biologic characteristics. The cancer stem cell hypothesis suggests that only a small subpopulation of cells, defined as cancer stem cells, has the ability to give rise to all of the cell types found in a particular cancer sample. Cancer stem cells play key roles in the tumorigenesis, growth, invasion, metastasis, recurrence and resistance to adjuvant therapy of cancer [22], [23]. Therefore, we suspect that low concentrations of metformin may affect pancreatic cancer stem cells and non-stem cancer cells differently, which could explain the drug’s *in vivo* anticancer action and the inconsistency of *in vitro* and *in vivo* results. Because CD133^+^ and CD24^+^CD44^+^ESA^+^ cells have been documented to be pancreatic cancer stem cells [24], [25], [26], we determined the effect of low concentrations of metformin on these subpopulations, demonstrating a decreased proportion of CD133^+^ cells. Considering the small effect of low concentrations of metformin on the proliferation of pancreatic cancer cells in general, it can be inferred that the proliferation of CD133^+^ cells, but not CD24^+^, CD44^+^, ESA^+^, or CD24^+^CD44^+^ESA^+^ cells, was selectively inhibited. Hermann et al. demonstrated that CD133^+^ and CD24^+^CD44^+^ESA^+^ cells overlapped but were not identical in L3.6pl cells derived from COLO 357 pancreatic cancer cells [25]. Our results showed that CD133^+^ cells but not CD24^+^CD44^+^ESA^+^ cells are detectable among AsPC-1 cells. As for SW1990 cells, CD24^+^CD44^+^ESA^+^ cells, which accounted for 5.46% of all cells, were insensitive to metformin, suggesting that the biological characteristics of CD133^+^ and CD24^+^CD44^+^ESA^+^ cells differ. We further determined the proportion of CD133^+^ cells every 24 h for 5 days after treatment of pancreatic cancer cells with low concentrations of metformin and produced growth curves for both CD133^+^ and CD133^–^ cells. The selective inhibition of CD133^+^ cells was clear. Accumulating *in vitro* evidence suggests that high concentrations of metformin may exert an antitumor effect by inducing apoptosis and/or cell cycle arrest, but there are few published data for low concentrations. Thus, we also investigated the effect of low concentration metformin on apoptosis and the cell cycle. Apoptosis, which has been documented to be important in the antitumor action of metformin, was induced in neither CD133^+^ nor CD133^–^ cells. G1/S arrest was induced in CD133^+^ but not in CD133^–^ cells. G1/S arrest may thus play a key role in the selective inhibition of CD133^+^ cells.

We then conducted *in vitro* and *in vivo* experiments to verify the anticancer action of low concentrations of metformin in pancreatic cancer. Pancreatic cancer cells treated with metformin for 72 h were used for cell invasion assays because the treatment decreased the proportion of CD133^+^ cells by half. Metformin was diluted in the drinking water of *nu*/*nu* mice 72 h before the xenograft experiment to achieve a steady state plasma concentration according to the pharmacokinetics of metformin [27]. Both the in vitro invasion assay and the in vivo xenograft assay supported the anticancer action of low concentration metformin. The differences of SW1990 xenograft tumor volumes were not statistically significant at day 21 (*P* = 0.062) or day 24 (*P* = 0.055). This may be due to the small sample size. Considering the small effect of metformin on the proliferation of CD133^–^ cells and the key role of cancer stem cells in tumor progression, invasion, and metastasis [28], [29], we tentatively suggest that selective inhibition of CD133^+^ cells contributes significantly to the in vitro and in vivo anticancer effect of metformin.

We next evaluated the expression of molecules that may determine the anticancer action of metformin on CD133^+^ pancreatic cancer cells. Activation of mTOR is regulated by growth factors and nutrients, and it regulates cell growth by controlling mRNA translation, ribosome biogenesis, autophagy, and metabolism [30]. Many targets of mTOR kinase are overexpressed or mutated in cancer, which correlates with cancer progression, adverse prognosis, and resistance to chemotherapy [31]. In recent years, mTOR was found to play important roles in maintaining cancer stem cells [32], [33]. Therefore, mTOR has been considered to be a therapeutic target in cancer that can be targeted by metformin [34], [35]. An inhibitory effect of metformin on the phosphorylation of mTOR, which has been reported in cancer cells including those of pancreatic cancer [36], [37], was observed in CD133^+^ pancreatic cancer cells in this study. Although metformin has been documented to induce activation of AMPK in pancreatic cancer cells, we did not observed this phenomenon, which may be due to the low concentration of metformin used in this study [36]. Erk and Akt are two other mediators of mTOR in cancer cells. Mutation of K-Ras, the predominant mutation in pancreatic cancer, leads to aberrant activation of Erk in pancreatic cancer cells, which in turn leads to mTOR activation [38]. We demonstrated concordance of the inhibitory activity of metformin on Erk and mTOR in CD133^+^ cells, which leads us to suggest that Erk dependent abrogation of mTOR activation plays an important role in the anticancer action of metformin. Recently, a similar selective inhibitory effect of metformin on CD133^+^ cancer cells due to metformin induced inhibition of Akt was documented in glioblastoma [39]. In contrast, our results demonstrate that metformin induced activation of Akt in CD133^+^ pancreatic cancer cells. We tentatively suggest that two mechanisms may contribute to the activation of Akt in CD133^+^ pancreatic cancer cells. First, metformin induces re-expression of miR-200 in pancreatic cancer [13]. FOG2 represses PI3K by binding the p85α subunit and miR-200 activates the PI3K/Akt signaling pathway by abrogating FOG2 [40]. Second, Erk dependent inhibition of mTOR may mediate a feedback loop that augments Akt phosphorylation [41].

In conclusion, our results reveal that low concentrations of metformin, of the same order as those measured in the plasma and tissues of individuals who have received a recommended therapeutic dose of metformin, selectively inhibits the proliferation of CD133^+^ pancreatic cancer cells and has an anticancer action both *in vitro* and *in vivo*. The antiproliferation effect of metformin on CD133^+^ pancreatic cancer cells may be due to Akt independent inhibition of mTOR phosphorylation. CD133^+^ pancreatic cancer cells are considered to be cancer stem cells that contribute to the recurrence, metastasis and resistance to adjuvant therapy of pancreatic cancer. Metformin is a widely used antidiabetic drug with limited side effects. These results provide a basis for the combination of metformin with current therapies to improve the prognosis of patients with pancreatic cancer.
